# Defect Engineering in Wüstite: Unlocking Control Over Iron Morphologies in Gas‐Solid Reduction

**DOI:** 10.1002/advs.202416713

**Published:** 2025-05-08

**Authors:** Qinghui Wu, Shuai Wang, Han Zhang, Fuchuan Zhang, Kaihui Ma, Jian Xu

**Affiliations:** ^1^ College of Materials Science and Engineering Chongqing University Chongqing 400044 China

**Keywords:** defect engineering, hydrogen‐based reduction, iron microstructure, sustainable steelmaking, wüstite lattice distortion

## Abstract

Hydrogen‐based direct reduction (HyDR) technology has emerged as a promising pathway for sustainable steelmaking. However, the efficiency and stability of HyDR are critically influenced by the microstructure evolution of iron during gas‐solid reduction reactions. Despite significant research on the reduction mechanisms of hydrogen (H_2_) and carbon monoxide (CO) with iron oxides, key aspects of the interplay between internal defects, pore dynamics, and reduction chemistry remain unresolved. In this study, the morphological evolution of iron during reduction with H_2_ and CO across a full concentration range at 900 °C is explored, establishing a direct link between lattice distortions in wüstite (Fe_x_O) and the resultant iron microstructure. Complementary analyses reveal that the concentration of defects in Fe_x_O governs these distortions. Specifically, low CO concentrations (< 80%) induce limited large‐scale defects, leading to single‐point nucleation and the growth of filament‐shaped iron whiskers. Conversely, H₂ and high CO concentrations (> 80%) create a high density of large‐scale defects, promoting multi‐point nucleation and the aggregation of tumor‐shaped iron structures. This work provides a multiscale perspective on how defect engineering in Fe_x_O modulates the morphologies of iron during reduction, offering valuable insights into optimizing reaction pathways to enhance efficiency and sustainability in materials processing.

## Graphical abstract

In this paper, a direct link between the concentration of defects in wüstite (Fe_x_O) and the resultant iron microstructure is established. By integrating the muti‐point nucleation and morphological evolution of iron at defect sites, a multiscale perspective on how defect engineering in Fe_x_O modulates the morphologies of iron has been developed.

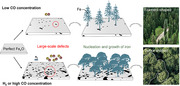

## 1 Introduction

Addressing climate change is a pressing global challenge, with carbon reduction emerging as both an economic priority and a societal imperative.^[^ ^1^ ^]^ The steel industry, one of the largest industrial contributors to greenhouse gas emissions, accounts for ≈6.5% of global carbon emissions and nearly 30% of manufacturing‐related emissions.^[^ ^2^, ^3^ ^]^ Under current production practices, cumulative carbon dioxide emissions from the steel industry are projected to reach 106.3 Gt between 2020 and 2050.^[^ ^4^ ^]^ The substantial environmental footprint primarily stems from the blast furnace ironmaking process, a fossil fuel–dependent method that accounts for 73.2% of global crude steel production.^[^ ^5^ ^]^ This underscores the urgent need for innovative ironmaking technologies that integrate clean energy solutions to achieve ultra‐low carbon steel production.^[^ ^6^, ^7^ ^]^ Emerging alternatives include plasma‐based methods,^[^ ^2^ ^]^ electrolysis,^[^ ^8^ ^]^ and gas‐based ironmaking processes.^[^ ^9^ ^]^

Among these alternatives, hydrogen‐based direct reduction (HyDR) technology has emerged as one of the most promising pathways for sustainable steelmaking. As the most mature option, HyDR accounts for over 72% of direct reduction iron production, relying on hydrogen‐rich fuels such as natural gas, coke oven gas, or pure hydrogen, to convert iron ores into sponge iron.^[^ ^1^, ^10^ ^]^ High temperatures enhance mass transfer and diffusion kinetics, facilitating continuous metallic iron precipitation during the reduction process. However, this process is complicated by the formation of filament‐shaped iron whiskers, which nucleate and grow directionally through complex interactions between reduction chemistry and evolving iron morphology. These iron whiskers, typically measuring 1–10 µm in length,^[^ ^11^ ^]^ tend to interconnect and aggregate, causing burden blockages that impair reactor performance.^[^ ^12^, ^13^ ^]^

Understanding and mitigating iron whiskers formation requires detailed insights into the multiscale mechanisms underlying the reduction process.^[^ ^14^, ^15^ ^]^ The HyDR process involves three sequential stages: hematite (Fe_2_O_3_) to magnetite (Fe_3_O_4_), Fe_3_O_4_ to wüstite (Fe_x_O), and Fe_x_O to metallic iron.^[^ ^16^, ^17^ ^]^ The final reduction step, which is the rate‐controlling step, serves as the primary stage for iron whiskers formation.^[^ ^18^, ^19^ ^]^ Early studies revealed that metallic iron growth occurred through a two‐stage mechanism: (1) nucleation upon reaching critical activation thresholds, followed by (2) competition between nucleation and diffusion rates.^[^ ^20^, ^21^ ^]^ Filament‐shaped whiskers formed when diffusion dominated, while dense iron structures developed when nucleation prevailed.^[^ ^22^ ^]^ The latter stage was influenced by multiple factors, including interfacial morphologies,^[^ ^23^ ^]^ reaction conditions,^[^ ^21^ ^]^ and rate‐controlling steps.^[^ ^24^ ^]^ This became the prevailing model for explaining iron whiskers formation during gas‐solid reduction. However, its fundamental limitation lied in its exclusive focus on the Fe_x_O to iron stage, neglecting critical differences in the preceding reduction pathways (Fe₂O₃→Fe_x_O) between hydrogen (H₂) and carbon monoxide (CO) environments. Consequently, it failed to provide consistent predictions across pure H₂, pure CO, and H₂‐CO mixed reducing conditions.^[^ ^25^ ^]^ To address this limitation, recent work had introduced mechanochemical models ^[^ ^11^, ^26^, ^27^, ^28^, ^29^ ^]^ that accounted for the cumulative effects of multi‐stage phase transformations during reduction. These models integrated three critical factors: lattice structure transformations,^[^ ^30^ ^]^ internal stress,^[^ ^31^ ^]^ and reaction kinetics.^[^ ^32^ ^]^ Notably, they successfully captured several key physical phenomena in microstructural evolution that were previously overlooked (e.g., the role of internal stress in driving the reduction). However, the morphological evolution of surface iron remained poorly characterized, especially its dynamic progression and underlying mechanisms.

Furthermore, several researchers had employed advanced experimental techniques and computational tools to elucidate the growth process of iron whiskers at finer scales. At the nanoscale, Zheng et al.^[^ ^13^ ^]^ observed the self‐assembly of iron nanoparticles into elongated grains, with dimensions approximately two orders of magnitude larger than the original particles. At the atomic scale, Lu et al.^[^ ^33^, ^34^ ^]^ utilized density functional theory calculations to reveal that CO exerted a directional force on iron ions, inducing preferential diffusion pathways that ultimately led to iron whiskers formation. Despite these advancements, existing models remained constrained by single‐scale descriptions, failing to comprehensively capture cross‐scale evolution processes, including defect evolution, lattice transformation, and iron nucleation/growth. This is because the complexities of an accurate process model stem from an intricate synergy of chemical, physical, and mechanical factors.^[^ ^35^ ^]^

To bridge this gap, we conduct a series of high‐temperature reduction experiments with pure Fe_2_O_3_ under H_2_ and CO atmosphere, complemented by molecular dynamics (MD) simulations. Our study is based on the new finding that the state of Fe_x_O plays a decisive role in determining iron morphologies at gas‐solid interfaces.^[^ ^11^, ^36^ ^]^ Using X‐ray diffraction (XRD) and high‐resolution transmission electron microscopy (HRTEM), we establish a direct correlation between Fe_x_O lattice distortion and the resulting iron morphologies. X‐ray photoelectron spectroscopy (XPS) and MD simulations confirm that high defect concentrations in Fe_x_O induce significant lattice distortions, inhibiting whisker formation under high CO concentrations or pure H_2_ at 900 °C. These insights provide a foundational framework for linking multiscale models and optimizing HyDR processes to improve efficiency and sustainability.

## 2 Results and Discussion

### 2.1 Iron Morphologies under Different Reduction Conditions

**Figure 1:**
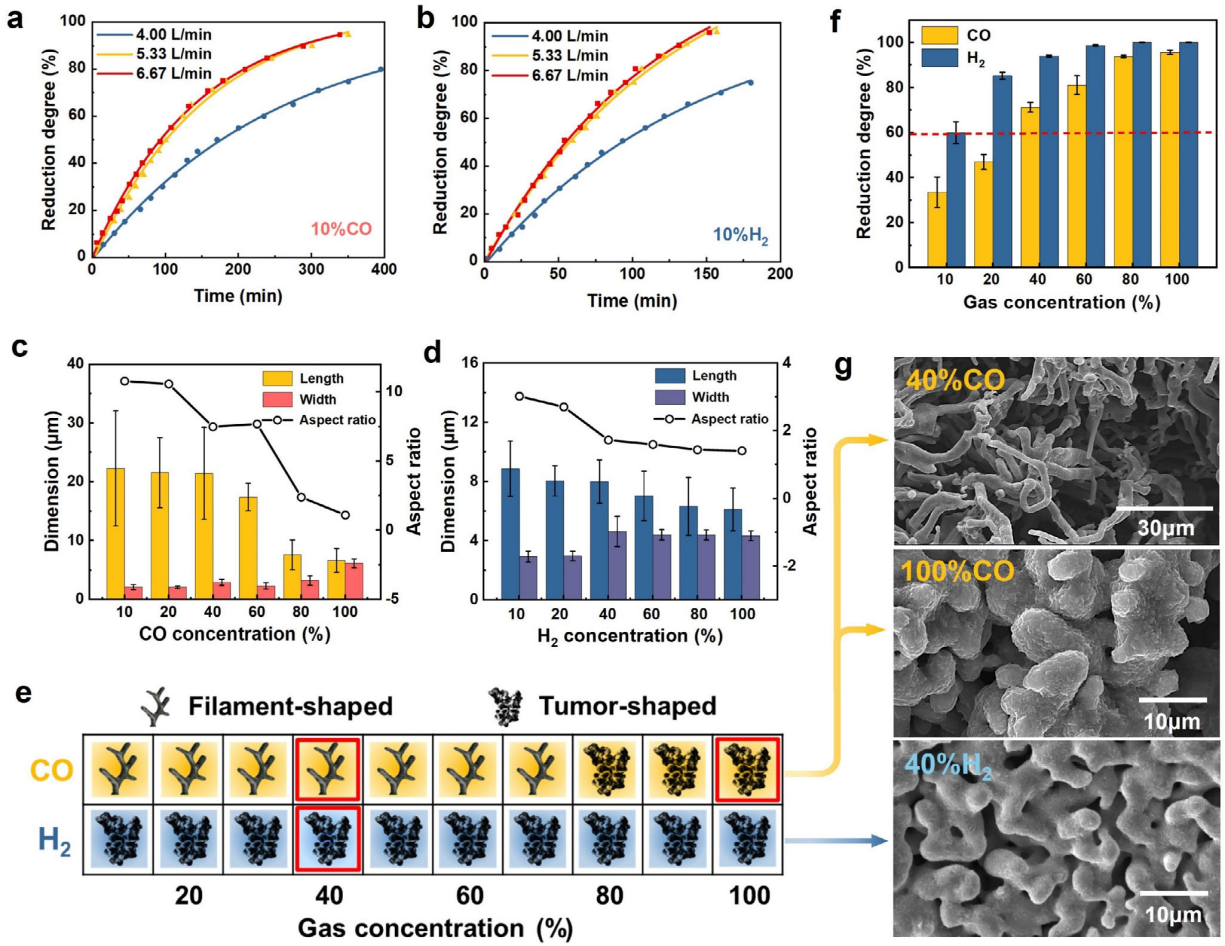
Iron morphologies under different reduction conditions. a, b) The reduction rate stabilizes when the gas flow rate exceeds 5.33 L min^−1^, indicating that external gas diffusion no longer limits the reaction, whether under 10% CO or 10% H₂. c, d) As the reducing gas concentration increases, the length of the newly formed iron decreases progressively, while its width increases. Notably, the morphological changes in the iron whiskers under CO are more pronounced. e) Two distinct iron whisker morphologies are defined based on the aspect ratio, and the results are summarized across all CO and H₂ concentrations. f) The reduction degree of the samples under varying CO and H₂ concentrations. g) SEM images showing filament‐shaped iron whiskers under 40% CO and tumor‐shaped iron under 100% CO and 40% H₂.

To eliminate the influence of external gas diffusion on the reduction results, preliminary experiments are conducted using Fe_2_O_3_ samples exposed to two distinct gas mixtures at 900 °C: 10% CO + 90% Nitrogen (N_2_) (in mole fraction, referred to as 10% CO), and 10% H₂ + 90% N_2_ (in mole fraction, referred to as 10% H₂). This temperature is chosen as it represents a typical operating condition for gas supply in HyDR processes. As shown in **Figure** **1**a,b, the reduction rate stabilizes when the gas flow rate exceeds 5.33 L min^−1^, indicating that external gas diffusion no longer limits the reaction. Based on this observation, a gas flow rate to 6.67 L min^−1^ is selected for all subsequent experiments to ensure that chemical reactions at the gas‐solid interface dominate during the initial reduction stage.

Fe_2_O_3_ samples are then subjected to reduction at 900 °C using reducing gas concentrations of 10%, 20%, 40%, 60%, 80%, and 100%, with CO and H_2_ serving as the reducing gas. Each reduction experiment lasts for 60 min. After reduction, the surface morphologies of the iron are characterized using scanning electron microscopy (SEM) (Figure S1d,e Supporting Information), and the statistical analysis of the iron morphology is presented in Figure 1c,d. The results indicate that as the reducing gas concentration increases, the length of the newly formed iron decreases progressively, while its width increases. These trends are consistent with the nucleation and diffusion competition theory,^[^ ^25^, ^37^ ^]^ which suggests that higher reducing gas concentrations enhance the reduction potential, significantly accelerating the nucleation rate. Under such conditions, multi‐site nucleation dominates, leading to radial iron growth. Interestingly, the sensitivity of iron morphology changes varies between CO and H_2_. When the CO concentration exceeds 80%, the iron morphology undergoes a significant transformation. The length of the iron decreases sharply from 17.37 to 7.52 µm, while its width increases from 2.26 to 3.16 µm. This indicates a shift in growth dynamics, with a notable reduction in the aspect ratio (length/width) of the iron. To quantify these morphological changes, the aspect ratio of the iron is used as a key metric. Iron microstructures with an aspect ratio below 4 are classified as tumor‐shaped, while those with an aspect ratio above 4 are categorized as filament‐shaped whiskers. Figure 1e summarizes the iron whisker morphologies across all reduction conditions.

The results clearly demonstrate that higher CO concentrations promote the transition from filament‐shaped whiskers to tumor‐shaped iron, highlighting the critical influence of gas concentration on iron morphology. Surprisingly, iron morphologies after reduction with H_2_ consistently appear as tumor‐shaped, even when the H_2_ concentration is as low as 10%. Based on weight loss calculations, the reduction degree of the 10% H₂ samples is 60.02%, significantly lower than those under 40% CO and 60% CO conditions, which exhibit filament‐shaped morphologies with reduction degree of 71.29% and 81.07%, respectively (Figure 1f). This suggests that the interfacial nucleation rate of 10% H₂ is significantly lower than that of 40% CO and 60% CO.

Moreover, dynamics are also strongly influenced by the diffusion of iron atoms, which is primarily temperature dependent. Previous studies have shown that temperature is the key factor governing the surface diffusion rate of iron atoms during the reduction of iron oxides by CO and H_2_.^[^ ^38^ ^]^ Matsumura ^[^ ^39^ ^]^ described the diffusion rate using the following equation at 912 °C.

(1) $$\;D_{s}=D_{i}\;exp\left(-\frac{E_{a}}{RT}\right)$$

where *D_i_* is the diffusion constant (2.4 m^2^ s^−1^), *E_a_* is the activation energy for diffusion (≈243 kJ mol^−1^), *R* is the gas constant (8.314 J mol^−1^ K^−1^), and *T* is the temperature in Kelvin.

According to the nucleation and diffusion competition theory, lower nucleation rates, such as those observed for 10% H₂, should favor the formation of filament‐shaped iron whiskers due to relatively enhanced surface diffusion. However, this expected behavior is not observed, instead, tumor‐shaped iron consistently forms under H_2_ reduction. This discrepancy highlights the limitations of the nucleation‐diffusion model in explaining the growth of iron whiskers under varying CO and H₂ conditions.

### 2.2 Correlation Between Fe_x_O Lattice Distortion and Iron Morphologies

**Figure 2:**
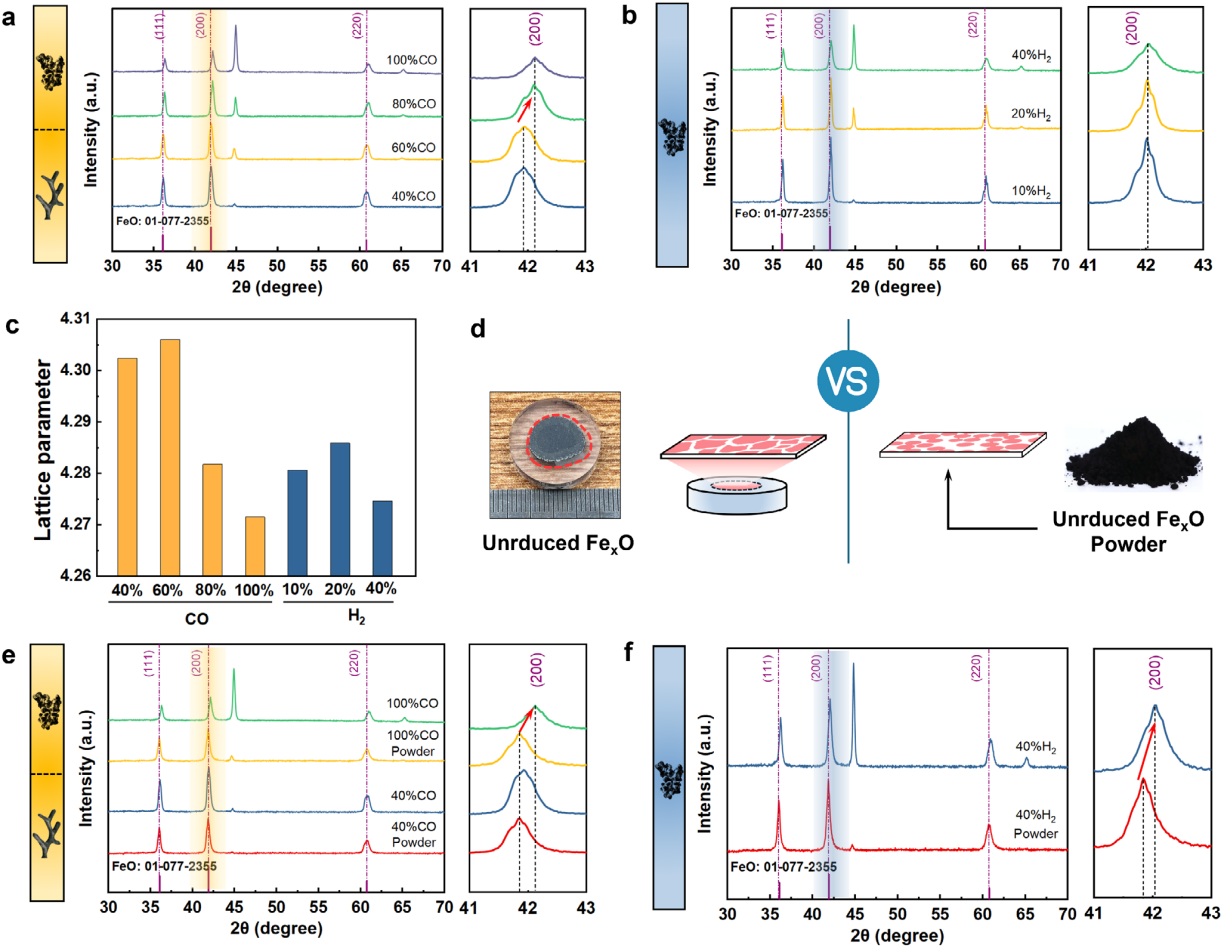
The crystallographic properties of unreduced Fe_x_O bulk and powder. a) Under conditions conducive to the tumor‐shaped iron (greater than 80% CO), the Fe_x_O peaks shift to higher angles. b) The characteristic peaks of Fe_x_O after H₂ reduction remain consistent across different concentrations. c) The lattice parameters of Fe_x_O bulk obtained through XRD refinement. d) A slow XRD scan of Fe_x_O powder is conducted and compared with those from Fe_x_O bulk. e) The XRD diffraction peak of Fe_x_O powder in a stress‐free state reveals Fe_x_O bulk under 100% CO reduction shifts toward higher angles, indicating significant lattice distortion. f) Similar lattice distortion is observed in Fe_x_O bulk under 40% H_2_ reduction.

To eliminate this limitation, we use XRD to examine the crystallographic properties of unreduced Fe_x_O in the preceding stage of iron formation. Under CO reduction conditions, the characteristic Fe_x_O peak corresponding to tumor‐shaped iron shows a significant shift to the right compared to that associated with filament‐shaped whiskers. Specifically, the strongest diffraction peak shifts from 41.92° to 42.10° (**Figure** **2a**). In contrast, The H_2_ reduction system reaches near‐complete conversion (reduction degree approaches 100%) when the concentration exceeds 60%. We specifically examine samples reduced under 10%, 20%, and 40% H₂. As expected, the characteristic Fe_x_O peaks across these samples consistently align, with the strongest peak appearing around 42.06° (Figure 2b). Additionally, the XRD peak at ≈45° corresponds to the diffraction of iron. As the reducing gas concentration increases, iron nuclei gradually form.

Further refinement of the XRD data reveals distinct differences in the lattice parameters of Fe_x_O under CO and H₂ conditions. When filament‐shaped whiskers form, Fe_x_O exhibits larger lattice parameters, around 4.306 Å. Conversely, during the formation of tumor‐shaped iron, the lattice parameters are smaller, ranging between 4.271 Å and 4.286 Å (Figure 2c, and the refined XRD details are presented in Figure S2, Supporting Information).

To investigate whether the observed changes in lattice parameters result from alterations in the coordination number of x in Fe_x_O ^[^ ^40^ ^]^ or lattice distortion caused by anisotropic swelling between grains during multiphase transformation, we prepare stress‐free Fe_x_O powder for detailed analysis.^[^ ^41^ ^]^ After removing the outer iron layer of the reduced sample, the exposed Fe_x_O core is ground into powder. Fine XRD scans are then performed on the powder to obtain crystallographic information (Figure 2d). The XRD analysis of stress‐free Fe_x_O powder reveals consistent peak positions for samples reduced under conditions favoring filament‐shaped whiskers (40% CO) and tumor‐shaped iron (100% CO), with the strongest diffraction peak at ≈41.85° (Figure 2e). XRD refinement confirms a lattice parameter of 4.308 Å for both conditions. However, the characteristic peaks of bulk Fe_x_O under 100% CO shift significantly to the right by ≈0.27°, compared to the stress‐free powder. This corresponds to a lattice distortion degree (Δd) of ≈0.036 Å, indicating significant lattice distortion during tumor‐shaped iron formation. In contrast, bulk Fe_x_O under 40% CO conditions exhibits minimal peak shift and negligible lattice distortion. Similar trends are observed under H_2_ reduction conditions producing tumor‐shaped iron (e.g., 40% H_2_ in Figure 2f), where the characteristic peaks of bulk Fe_x_O shift to the right by ≈0.22°, resulting in a distortion degree (Δd) of ≈0.033 Å. These findings confirm that lattice distortion is a key structural feature of Fe_x_O during tumor‐shaped iron formation. Detailed XRD refinement results for stress‐free Fe_x_O powder are provided in Figure S3 (Supporting Information).

**Figure 3:**
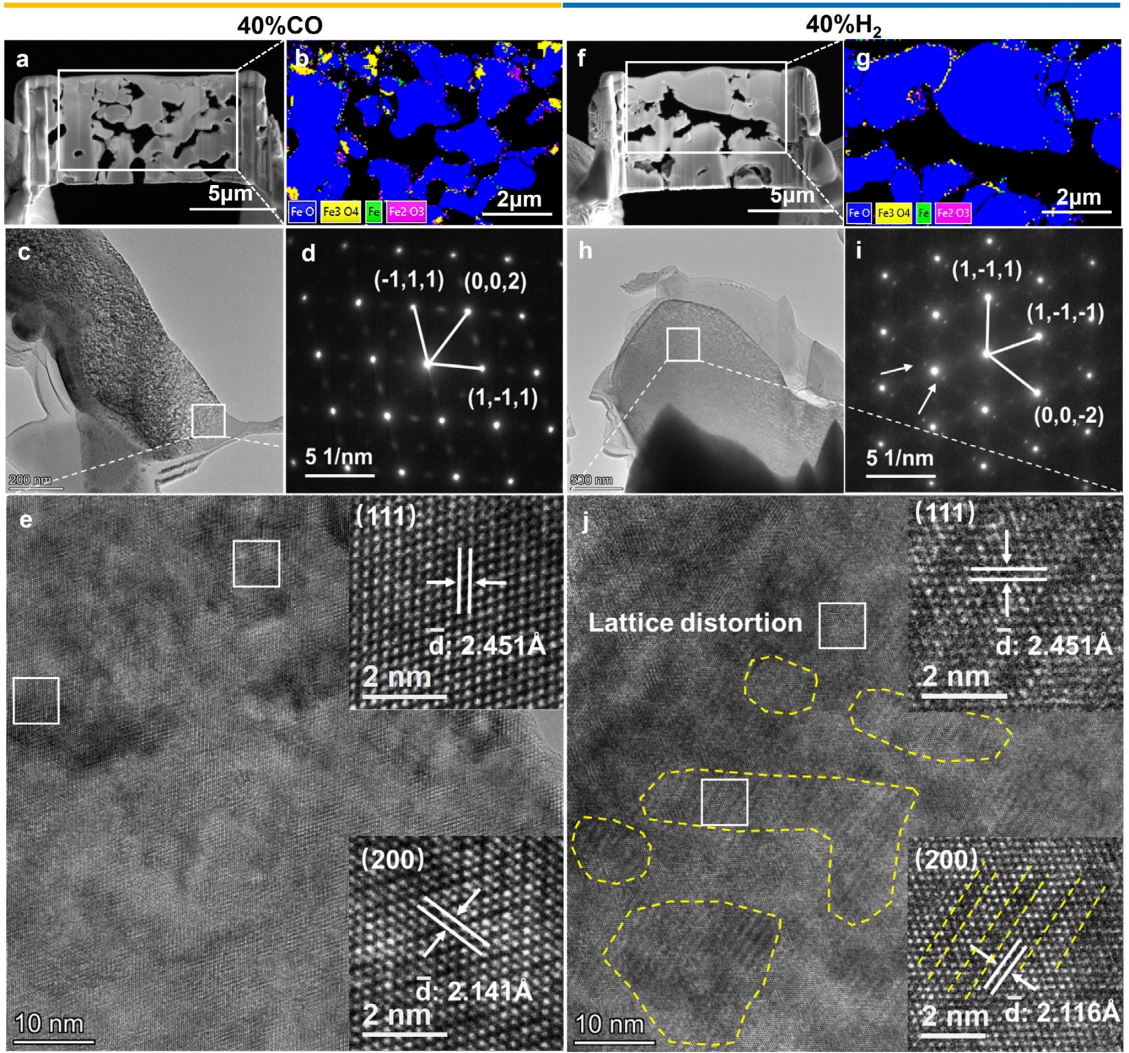
HRTEM results. a) Fe_x_O reduced under 40% CO conditions is sectioned and thinned to 10 nm using FIB. b) Backscattered electron diffraction confirms Fe_x_O as the primary phase. c) The morphology of Fe_x_O observed under HRTEM. d) A 70 nm^2^ selected‐area electron diffraction pattern. e) Statistical analysis of 10 randomly selected 5 nm^2^ areas reveals lattice spacings of 2.451 Å on the (111) plane and 2.141 Å on the (200) plane. f,g) Similarly, FIB is used to thin Fe_x_O reduced under 40% H₂ conditions to 10 nm. h) The morphology of Fe_x_O observed under HRTEM. i) A 70 nm^2^ selected‐area electron diffraction pattern shows distinct satellite spots. j) Statistical analysis indicates a reduction in lattice spacing along the (200) plane to 2.116 Å, and this lattice distortion is prevalent in Fe_x_O reduced by 40% H₂.

To further investigate lattice distortion in Fe_x_O, HRTEM is performed on Fe_x_O samples reduced under 40% CO and 40% H₂ conditions. Thin sections (≈10 nm) are prepared using focused ion beam (FIB) with tungsten plating to minimize ion beam erosion (Figure S4, Supporting Information). Backscattered electron diffraction confirms that Fe_x_O is the primary phase, with minimal oxidation to Fe_3_O_4_ at the edges (**Figure** **3**b,g). Selected‐area electron diffraction (SAED) analysis of two 70 nm^2^ Fe_x_O regions (Figure 3c,h) confirms their orientation along the [110] zone axis (Figure 3d,i). Notably, the SAED patterns of Fe_x_O reduced under 40% H₂ exhibit additional satellite diffraction spots, indicating the presence of a periodic modulation structure with [002] propagation direction. These modulation characteristics are associated with oxygen vacancy ordering (see Section 2.3) and further verified by HRTEM. Statistical analysis of lattice spacings across ten randomly selected 5 nm^2^ regions reveals two distinct values for the 40% CO‐reduced Fe_x_O: 2.451 Å corresponding to the (111) planes and 2.141 Å for the (200) planes (Figure S5a,b, Supporting Information; Figure 3e). In contrast, for Fe_x_O reduced under 40% H₂, the lattice spacing along the (111) planes remains consistent at 2.451 Å (Figure S5c, Supporting Information), but the spacing along the (200) planes decreases to 2.116 Å (Figure S5d, Supporting Information). This pronounced lattice distortion along the (200) planes generates prominent moiré fringes, which are consistently observed in HRTEM images (Figure 3j).

SEM analysis of the unreduced Fe_x_O microstructure provides additional evidence of the role of intergranular compression in lattice distortion. Under conditions favoring filament‐shaped whiskers (40% CO), Fe_x_O grains appear loosely distributed with visible intergranular pores (Figure S6a, Supporting Information). In contrast, samples reduced under conditions favoring tumor‐shaped iron (100% CO and 40% H₂) exhibit a dense structure, with tightly compressed Fe_x_O grains and nearly eliminated intergranular pores (Figure S6b,c, Supporting Information). This compression of micrometer‐scale grains likely contributes to the observed lattice distortion.

### 2.3 The Role of Oxygen Vacancy Concentration in Lattice Distortion

**Figure 4:**
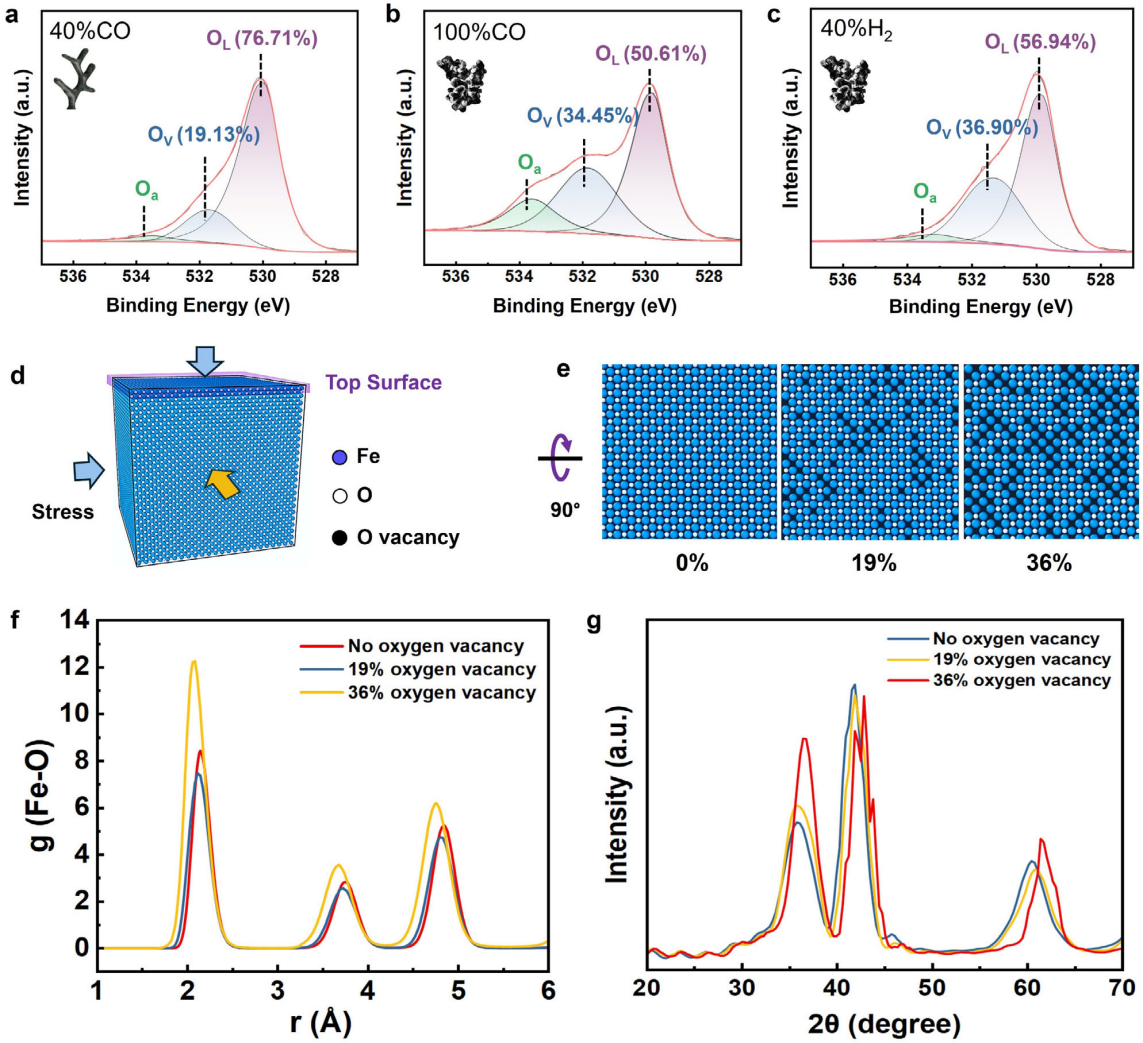
a‐c) The O 1s XPS spectrum of Fe_x_O. (a) Fe_x_O displays a low O_v_ concentration when reduced in 40% CO condition, while (b) 100% CO and (c) 40% H₂ conditions lead to a significantly higher O_v_ concentration. d‐g) MD simulations of oxygen vacancy defects in Fe_x_O. d) A complete Fe_x_O crystal model containing ≈ 100000 atoms. (e) Three Fe_x_O models with varying O_v_ concentrations (0%, 19%, and 36%). (f) Increasing the O_v_ from 19% to 36% leads to a significant reduction in Fe‐O bond length. (g) Simulated XRD patterns show a pronounced peak shift toward higher angles with increasing O_v_ concentrations.

To elucidate the origin of lattice distortion in Fe_x_O, we employ XPS to examine its molecular structure. The deconvoluted O 1s XPS spectrum reveals three distinct peaks at 529.97 eV, 531.70 eV, and 533.50 eV, corresponding to lattice oxygen (O_L_), oxygen vacancies (O_v_), and surface‐adsorbed oxygen species (O_a_), respectively.^[^ ^42^, ^43^ ^]^ The O_v_ peak at 531.70 eV represents vacancies created by the extraction of lattice oxygen atoms during reduction. As shown in **Figure** **4**a‐c, Fe_x_O samples reduced under conditions favoring tumor‐shaped iron exhibit a significantly high O_v_ concentration (≈ 36%) compared to those forming filament‐shaped whiskers (≈ 19%).

MD simulations are conducted to further investigate the impact of O_v_ concentration on lattice distortion. Three cubic Fe_x_O crystal models (100 Å × 100 Å × 100 Å) with O_v_ concentrations of 0%, 19%, and 36% are constructed (Figure 4d‐e). The models are equilibrated in the NPT ensemble at 900 °C and 1 bar for 20 ns, then cooled to room temperature at 4.365 °C ns^−1^. Radial distribution functions (RDF) analysis reveals a decrease in Fe‐O bond length from 2.15 to 2.05 Å (Figure 4f) as O_v_ concentration increases from 19% to 36%, indicating significant lattice distortion. Simulated XRD diffraction patterns further confirm this effect, showing a rightward peak shift from 41.84° to 42.8° as O_v_ concentrations increase (Figure 4g). This shift aligns with experimental observations of bulk Fe_x_O under conditions favoring tumor‐shaped iron formation.

### 2.4 Mechanistic Model for Morphological Evolution at the Gas‐Solid Interface

**Figure 5:**
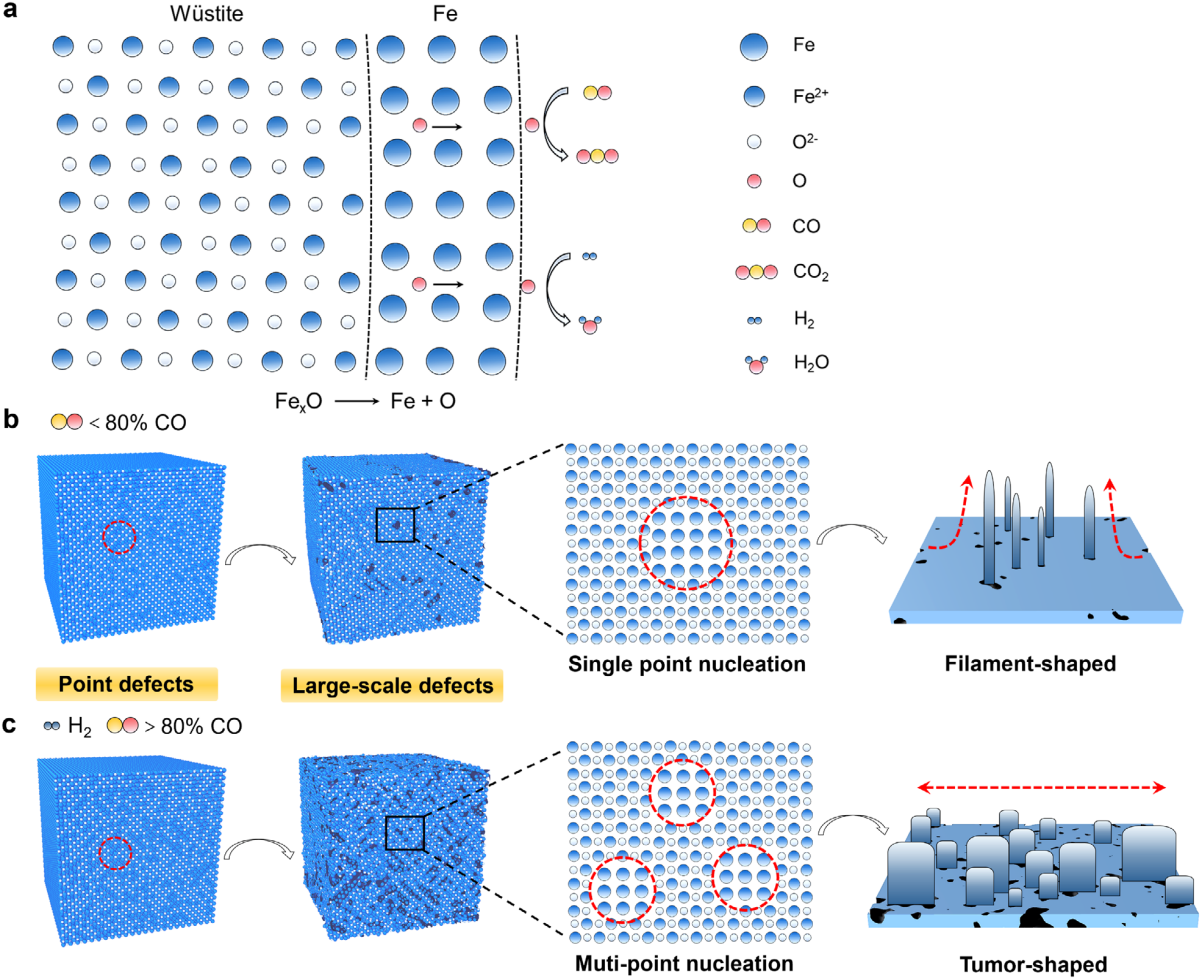
A mechanistic model illustrates the influence of defect concentrations on iron morphologies. a) The transformation of Fe_x_O to Fe is driven by the depletion of sub‐stoichiometric oxygen. b) In low‐defect Fe_x_O (formed under < 80% CO), limited large‐scale defects result in single‐point nucleation, promoting filament‐shaped whiskers growth. c) Under high CO (> 80%) and H_2_, Fe_x_O develops a high density of large‐scale defects, facilitating multi‐point nucleation and leading to tumor‐shaped iron formation.

We propose a mechanistic model linking O_v_ concentrations to iron morphologies. During reduction, CO and H₂ create point defects, primarily oxygen vacancies, as lattice oxygen is extracted (**Figure** **5a**).^[^ ^44^, ^45^ ^]^ At low CO concentrations (< 80%), Fe_x_O forms with a relatively low O_v_ concentration (≈ 19%), resulting in minimal large‐scale defects during the phase transition (Figure 5b). In contrast, high CO concentrations (> 80%) and H₂ induce a high O_v_ concentration (≈ 36%), leading to large‐scale defects such as edge dislocations, discontinuous lattice stripes, and lattice distortions ^[^ ^46^, ^47^ ^]^ (Figure 5c).

Iron nucleation is driven by the diffusion of sub‐stoichiometric oxygen toward the gas‐solid interfaces. Moreover, Large‐scale defect sites, characterized by less stable atomic arrangements, exhibit higher energy and are more reactive, making them preferential sites for nucleation. In Fe_x_O with low defect density, nucleation occurs at isolated points, promoting directional growth and the formation of filament‐shaped whiskers. In defect‐rich Fe_x_O, the high density of large‐scale defects facilitates multi‐point nucleation, leading to iron aggregation and tumor‐shaped morphologies.

## 3 Conclusion

The ultra‐low‐carbon transition in the steel industry is vital for reducing greenhouse gas emissions. Given the blast furnace's reliance on carbon, HyDR ironmaking has become a key focus. However, a critical challenge is the sticking of furnace burden, primarily caused by iron whiskers formation. To address this, we investigate the iron morphology evolution under full‐concentration CO and H₂ atmosphere at 900 °C.

Our results show that higher CO concentrations promote the transition from filament‐shaped whiskers to tumor‐shaped iron, whereas H₂ consistently yields tumor‐shaped morphologies. Lattice distortion, measured at ≈0.036 Å, is a key structural feature of Fe_x_O during tumor‐shaped iron formation and is closely linked to defect concentration. Specifically, low CO concentrations (< 80%) result in an O_v_ concentration of ≈ 19%, leading to fewer large‐scale defects and promoting single‐point nucleation for whisker growth. In contrast, high CO concentrations (> 80%) and H₂ induce an O_v_ concentration of ≈ 36%, creating a high density of large‐scale defects that facilitate multi‐point nucleation and tumor‐like aggregation.

Our findings emphasize the critical role of Fe_x_O's crystal structures in determining iron morphologies. These insights provide a foundation for optimizing chemical and mass transfer models, and designing advanced HyDR ironmaking processes tailored for industrial applications.

## 4 Experimental Section

### Input Material and Reduction Procedure

High‐purity Fe_2_O_3_ powder (Fe_2_O_3_ ≥ 99.9%) with a median particle size of 1.748 µm (Figure S7, Supporting Information) is used as raw material. SEM characterization confirms a quasi‐spherical morphology (Figure S1a, Supporting Information). Approximately 10 g of the powder is pressed into cylindrical pellets (≈20 mm in diameter and ≈12 mm in height) under 20 kPa pressure for 30 s (Figure S8a, Supporting Information). Three cylindrical samples are simultaneously heated to 900 °C at a heating rate of 10 °C min^−1^. SEM analysis shows a dense, planar surface morphology with minor porosity due to the compaction and solid‐state sintering (Figure S1b, Supporting Information). The samples are then maintained at 900 °C for 60 min under reducing atmosphere with gas composition detailed in Figure S8c (Supporting Information). The reduction process employs a reducing gas flow rate of 6.67 L min^−1^. After reduction, the samples are furnace‐cooled under N_2_ at 2 L min^−1^ to prevent oxidation (Figure S9, Supporting Information). The reduction degree is calculated using the following equation:

(2) $$\mathrm{redu}\mathrm{ctio}n\;\deg\mathrm{ree}=\frac{m_{0}-m_{t}}{0.3\cdot m_{0}}\;$$

where, *m*_0_ is the the initial sample mass (g), and *m_t_* is the mass (g) at time *t* (min).

### Microstructural Characteristics

The morphology of reduced samples at the gas‐solid interface is analyzed using high‐resolution SEM (TESCAN, VEGA3) at an accelerating voltage of 20 kV. Consistent with the unreacted core model for gas‐solid reduction,^[^ ^48^ ^]^ the outer layer of the reduced sample consists of metallic iron, while the core remains as unreacted Fe_x_O. To examine the Fe_x_O core, the metallic iron layer is carefully removed, and the exposed Fe_x_O is embedded in epoxy resin. Once solidified, the embedded samples are mechanically sanded and polished with a 0.25 µm diamond spray to prepare cross‐sections. Samples are then vacuum‐dried at 80 °C to prevent oxidation. XRD (MALVERN PANALYTICAL, Aeris) with a scanning rate of 1 ° min^−1^ is used to analyze compositional and crystallographic properties. SEM further examines the internal microstructure of Fe_x_O pellets.

For atomic‐scale characterization, thin slices of Fe_x_O (< 10 nm) are prepared using a FIB (FEI, Helios 5 CX) with tungsten plating protection to minimize oxidation. These slices are then analyzed using HRTEM (FEI, Talos F200S) to investigate crystal structures.

### Molecular Dynamic Simulations

MD simulations are performed using the LAMMPS package.^[^ ^49^ ^]^ The Fe_x_O model is constructed based on the experimental crystallographic data,^[^ ^50^ ^]^ and its accuracy is validated by comparing simulated XRD patterns with a Cu‐Kα target (λ = 0.154 nm) (Figure S10a, Supporting Information). The interactions between Fe and O atoms are modeled using the Analytical Bond‐Order Potential (ABOP),^[^ ^51^ ^]^ which incorporates the Tersoff potential with short‐range interaction corrections via the Ziegler‐Biersack‐Littmark shielding potential. The total system energy is given by:

(3) $$E=\sum_{i}\sum_{j>i}E_{ij}=\sum_{i}\sum_{j>i}f_{C}\left(r_{ij}\right)\left[E_{R}\left(r_{ij}\right)-\bar{b_{ij}}E_{A}\left(r_{ij}\right)\right]$$

where, *E_R_* (*r_ij_*) and *E_A_* (*r_ij_*) represent the repulsive and attractive Morse‐like potentials, *f_C_* (*r_ij_*) is the interaction cutoff function, and $\bar{b_{ij}}$ is the bond‐order function accounting for many‐body effects.

The accuracy of ABOP is validated through heating simulations in the NVT ensemble, with a heating rate of 34 °C ps^−1^ over 50 ps. The simulation predicts a critical potential energy transition point at 1369 °C (Figure S10b, Supporting Information), closely aligning with the experimentally measured melting point of Fe_x_O, confirming the force field's reliability.

## Conflict of Interest

The authors declare no conflict of interest.

## Supporting information



Supporting Information

## Data Availability

The data that support the findings of this study are available from the corresponding author upon reasonable request.
