# Supplementary material for: Defect Engineering in Wüstite: Unlocking Control Over Iron Morphologies in Gas‐Solid Reduction
Source: Adv Sci (Weinh). 2025 May 8;12(22):2416713. doi: 10.1002/advs.202416713 (PMC12165032; doi:10.1002/advs.202416713)
Supplement: Supplementary file 1 — Supporting Information [file ADVS-12-2416713-s001.docx]

**Defect Engineering in Wüstite: Unlocking Control Over Iron Morphologies in Gas-Solid Reduction**

Qinghui Wu, Shuai Wang, Han Zhang, Fuchuan Zhang, Kaihui Ma, Jian Xu*

College of Materials Science and Engineering, Chongqing University, Chongqing, 400044, China

*Jian Xu

**Email:**  [jxu@cqu.edu.cn](mailto:jxu@cqu.edu.cn)

**Keywords:** Defect engineering; Wüstite lattice distortion; Iron microstructure; Hydrogen-based reduction; Sustainable steelmaking

**This file includes:**

Figures S1 to S10


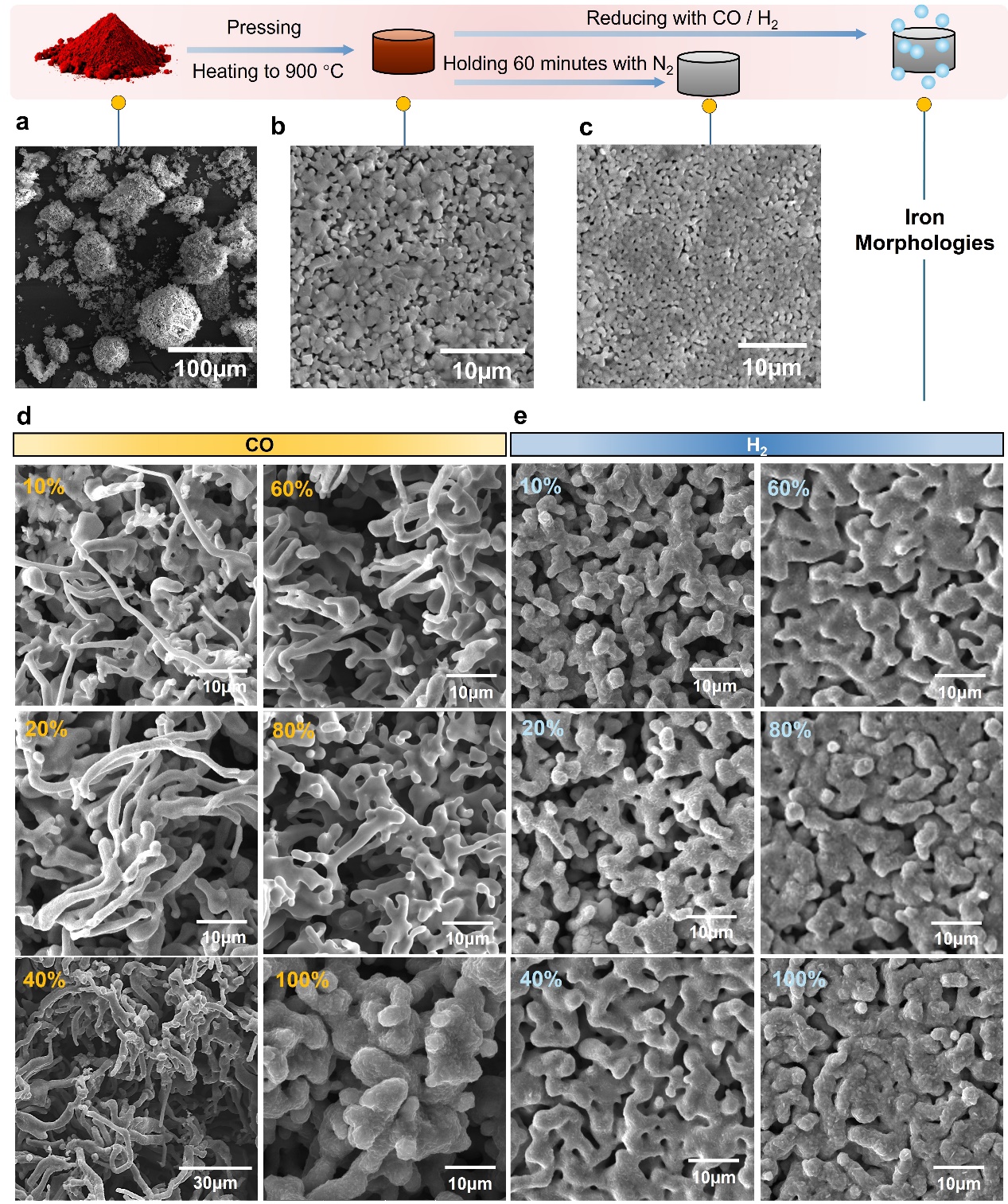


Fig. S1. SEM images of (a) Fe_2_O_3_ powder, (b) Fe_2_O_3_ pellet before reduction and (c) Fe₂O₃ pellets maintained with N_2_ for 60 minutes. (d, e) Filament-shaped iron whiskers formed after reduction in low CO concentrations (< 80%CO), and tumor-shaped iron whiskers formed in high CO (> 80%CO) and H₂ conditions.


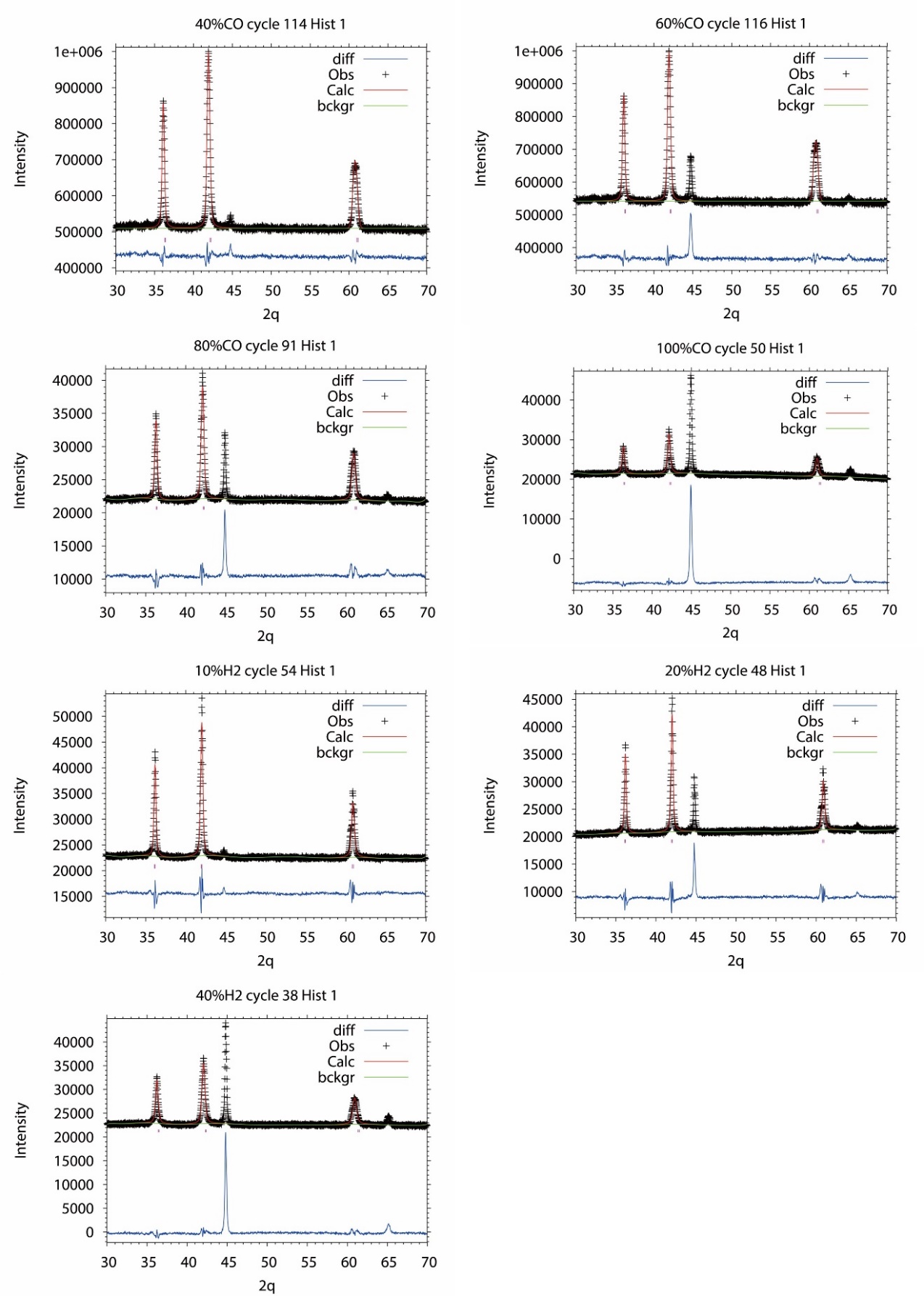


Fig. S2. XRD refinement details of unreduced Fe_x_O.


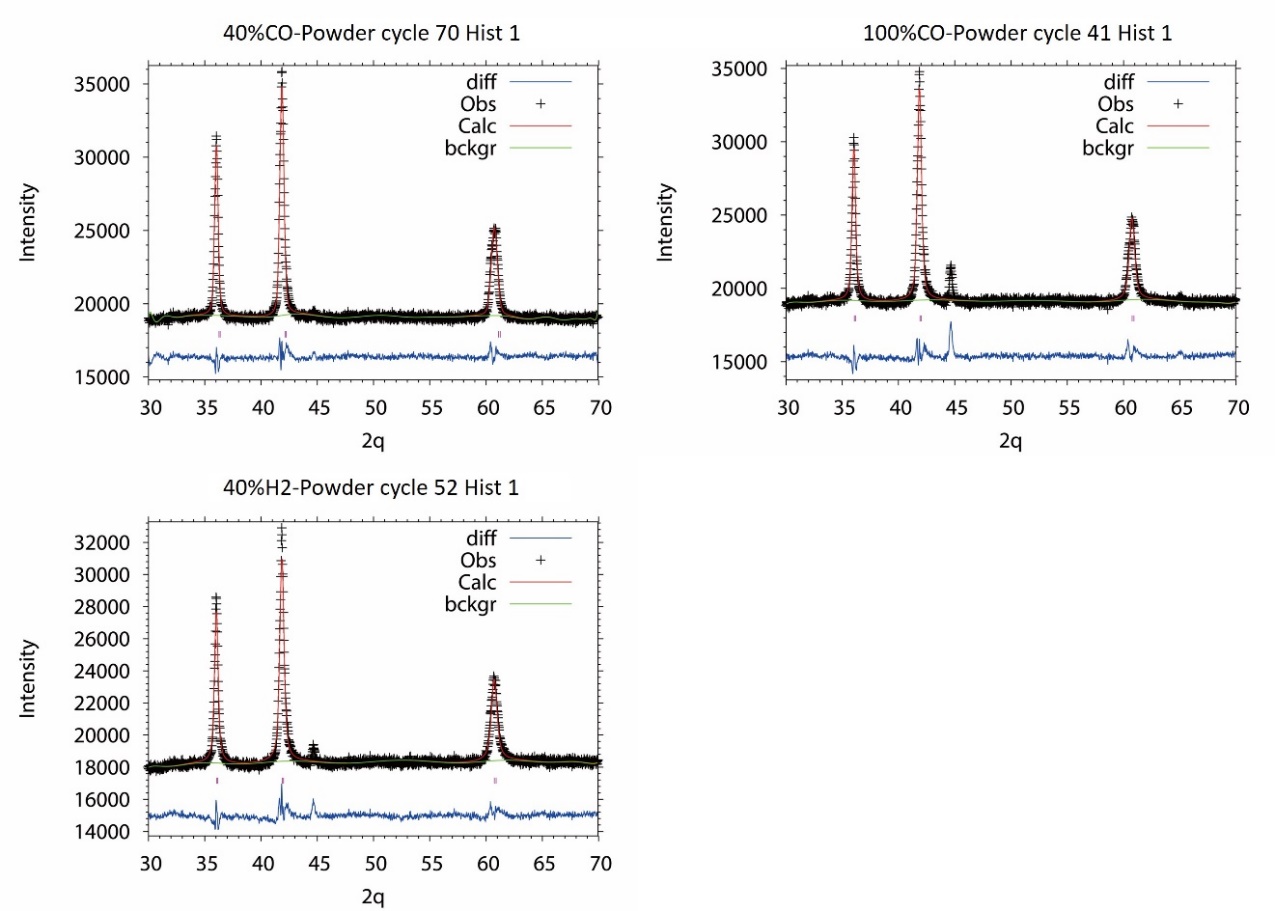


Fig. S3. XRD refinement details of Fe_x_O powder.


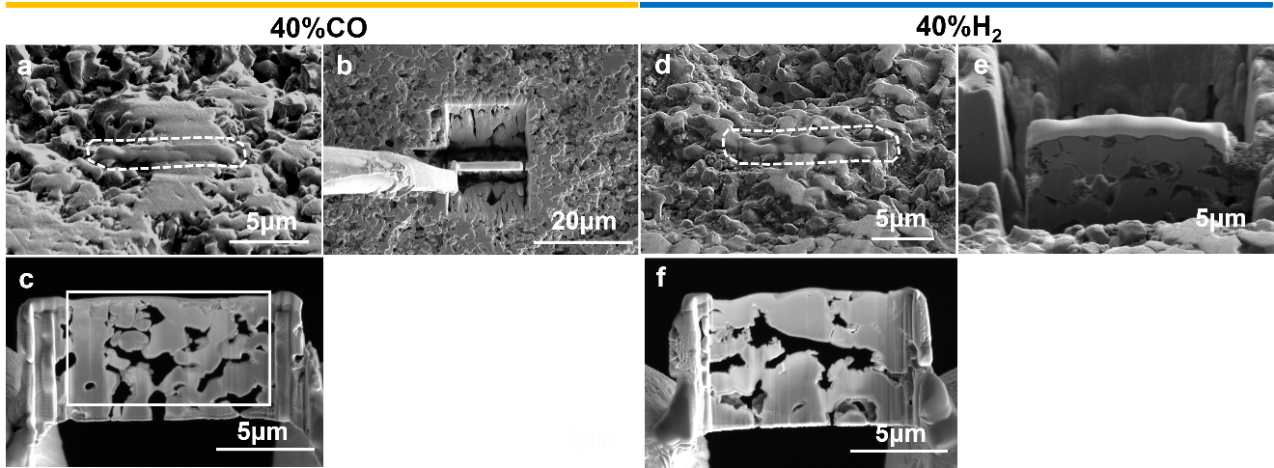


Fig. S4. FIB-prepared thin sections (~ 10 nm) of Fe_x_O bulks samples reduced under (a-c) 40% CO and (d-f) 40%H_2_ conditions.


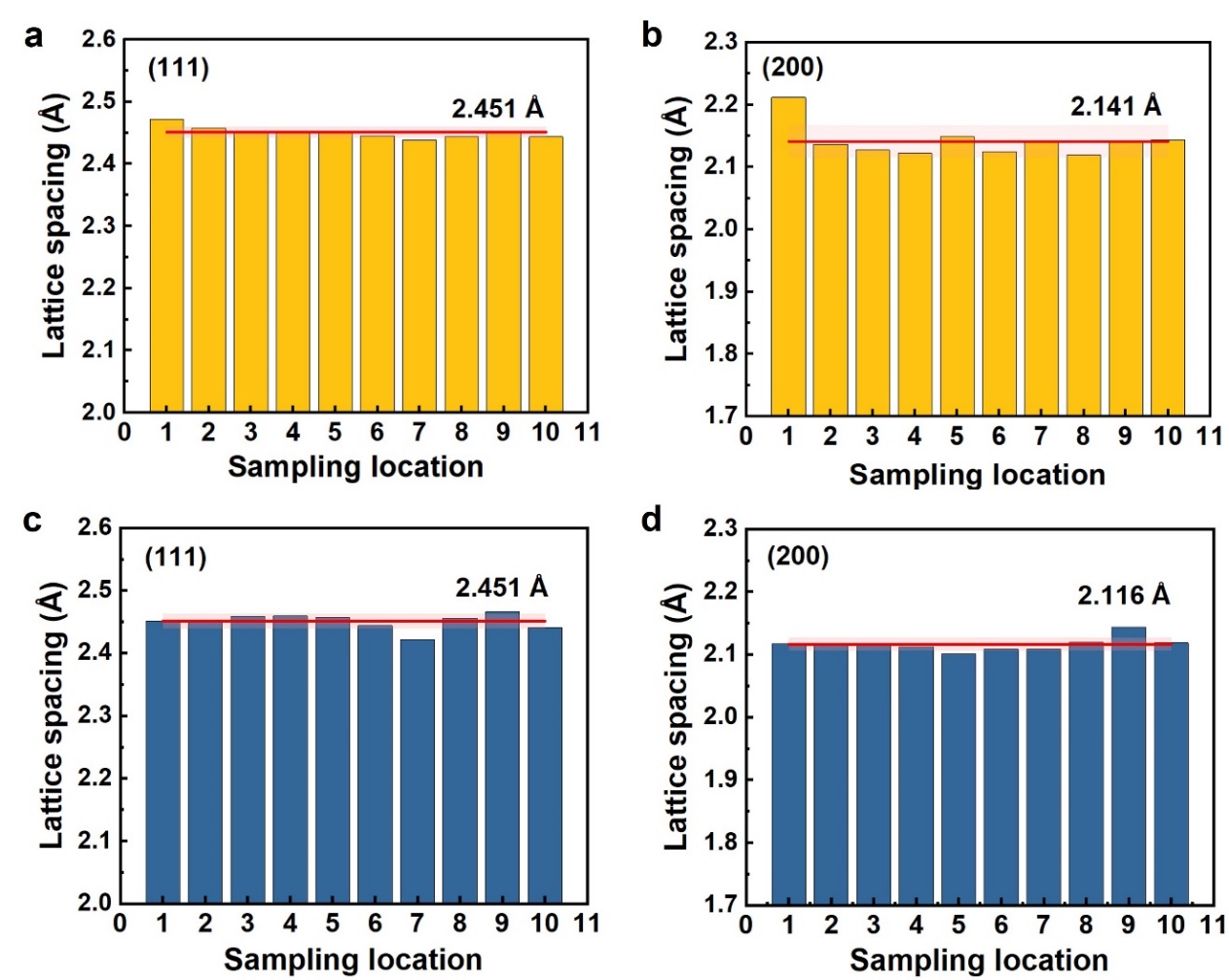


Fig. S5. Statistical analysis of lattice spacing. (a) (111) plane of Fe_x_O reduced by 40% CO. (b) (200) plane of Fe_x_O reduced by 40% CO. (c) (111) plane of Fe_x_O reduced by 40% H_2_. (d) (200) plane of Fe_x_O reduced by 40% H_2_.


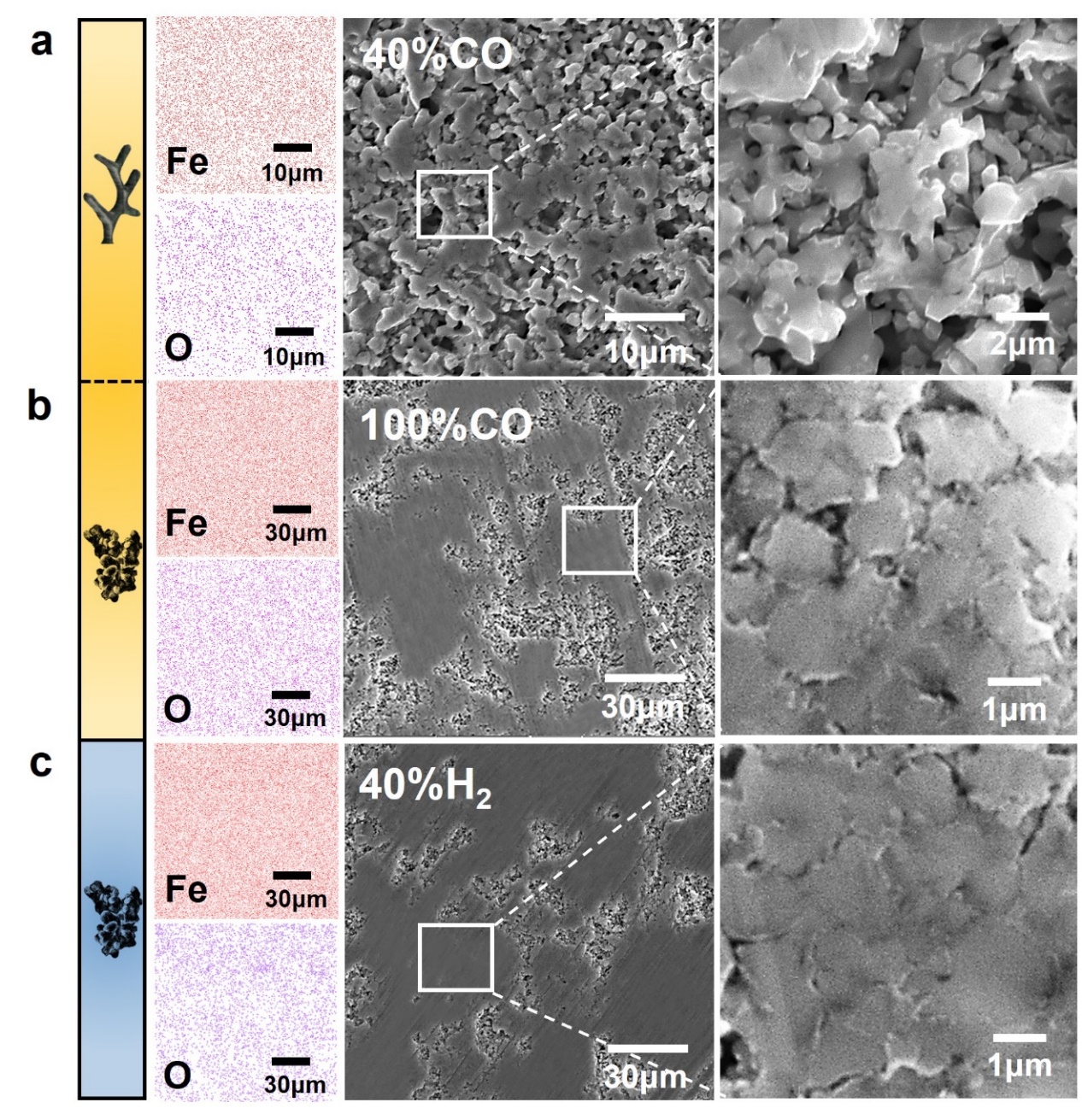


Fig. S6. Microstructure of unreduced Fe_x_O. (a) Loosely distributed Fe_x_O grains with intergranular pores after reduction in 40% CO. (b, c) Densified structures observed in samples reduced under conditions favoring tumor-shaped iron formation.

**
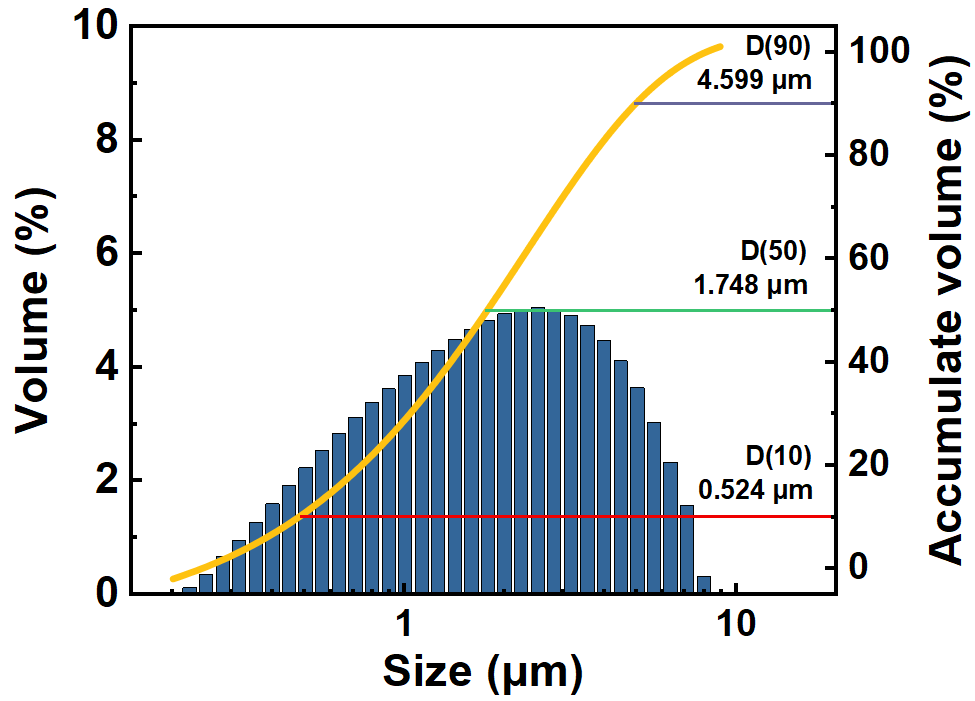
**

Fig. S7. Particle size distribution of pure ferric oxide powder


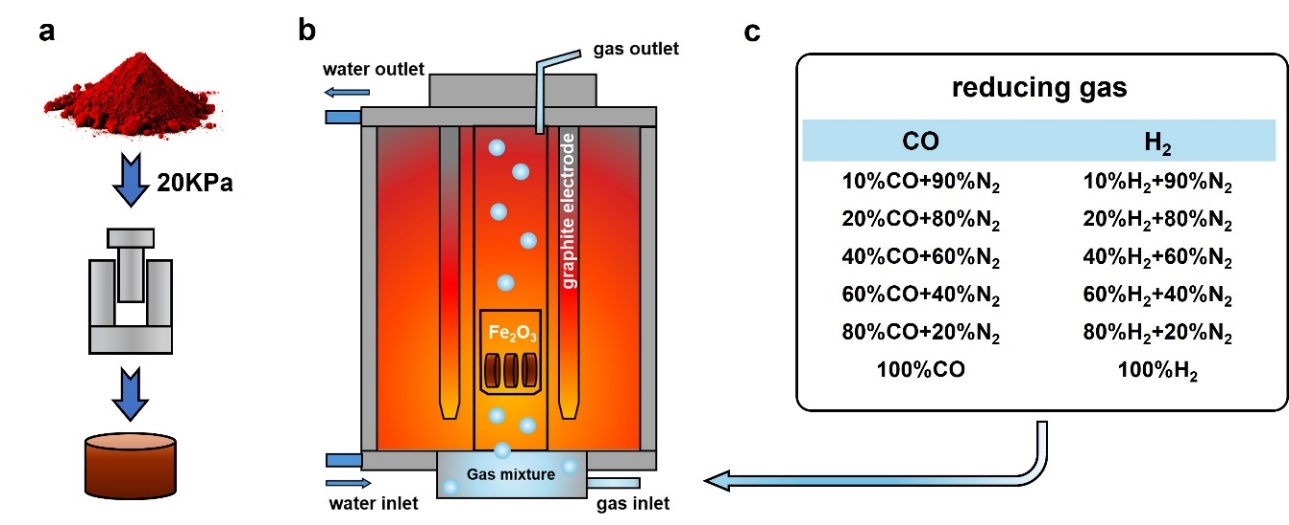


Fig. S8. Schematic of high-temperature reduction experiments. (a) Compression of Fe_2_O_3_ powder into cylindrical pellets under 20 kPa. (b) Isothermal reduction of three samples at

900 °C. (c) Composition of reducing gas used in the process.

**
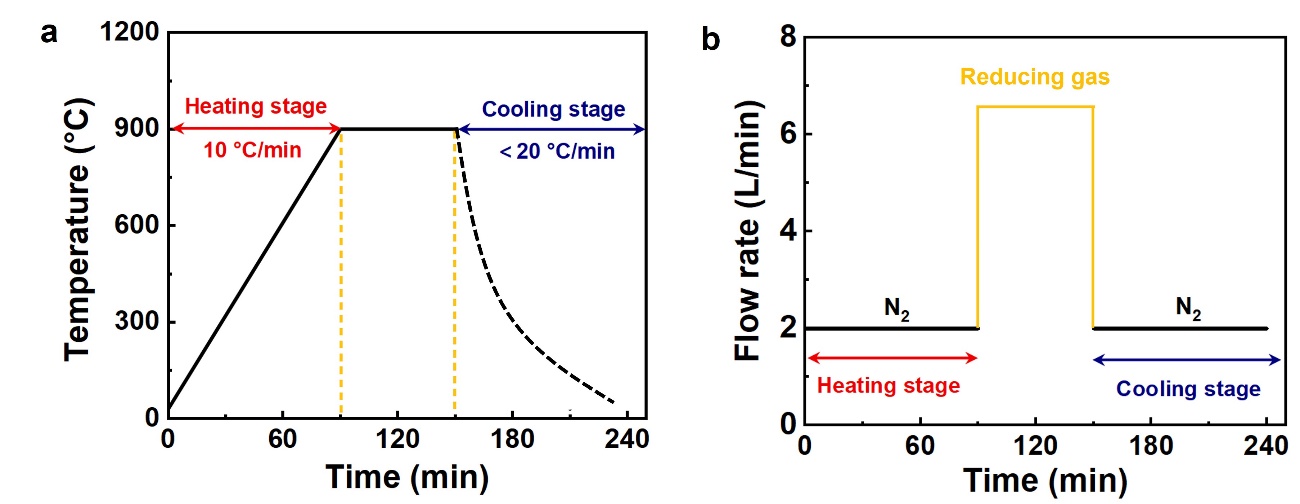
**

Fig. S9. (a) Heating and cooling curves and (b) gas flow rates during reduction experiments.


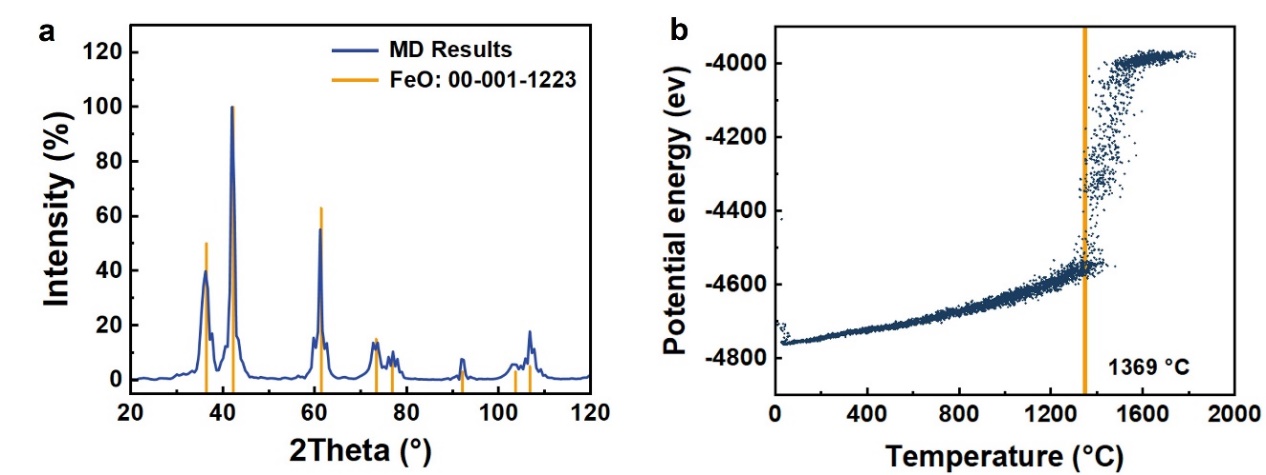


Fig. S10. Validations of the FeO model and force field for MD simulation. (a) XRD analysis of FeO model using a Cu-Kα target. (b) Potential energy evolution of the FeO system with the applied force field.
